# Recognition of Impulse of Love at First Sight Based On Photoplethysmography Signal

**DOI:** 10.3390/s20226572

**Published:** 2020-11-17

**Authors:** Huan Lu, Guangjie Yuan, Jin Zhang, Guangyuan Liu

**Affiliations:** 1 College of Electronic and Information Engineering, Southwest University, Chongqing 400715, China; luhuan@email.swu.edu.cn (H.L.); gjy000358@email.swu.edu.cn (G.Y.); zhangjin1995@email.swu.edu.cn (J.Z.); 2 Institute of Affective Computing and Information Processing, Southwest University, Chongqing 400715, China; 3 Chongqing Key Laboratory of Nonlinear Circuits and Intelligent Information Processing, Southwest University, Chongqing 400715, China

**Keywords:** impulse of love at first sight, PPG, feature selection, machine learning

## Abstract

Love at first sight is a well-known and interesting phenomenon, and denotes the strong attraction to a person of the opposite sex when first meeting. As far as we know, there are no studies on the changes in physiological signals between the opposite sexes when this phenomenon occurs. Although privacy is involved, knowing how attractive a partner is may be beneficial to building a future relationship in an open society where both men and women accept each other. Therefore, this study adopts the photoplethysmography (PPG) signal acquisition method (already applied in wearable devices) to collect signals that are beneficial for utilizing the results of the analysis. In particular, this study proposes a love pulse signal recognition algorithm based on a PPG signal. First, given the high correlation between the impulse signals of love at first sight and those for physical attractiveness, photos of people with different levels of attractiveness are used to induce real emotions. Then, the PPG signal is analyzed in the time, frequency, and nonlinear domains, respectively, in order to extract its physiological characteristics. Finally, we propose the use of a variety of machine learning techniques (support vector machine (SVM), random forest (RF), linear discriminant analysis (LDA), and extreme gradient enhancement (XGBoost)) for identifying the impulsive states of love, with or without feature selection. The results show that the XGBoost classifier has the highest classification accuracy (71.09%) when using the feature selection.

## 1. Introduction

The impulse of love at first sight (ILFS) is not a distinct form of love, but rather represents a strong initial attraction [1]. In real life, the ILFS is a well-known phenomenon. The research of Alea et al. [2] showed that ILFS affects the quality of a relationship, as memories established early in the relationship often remain influential in the later stages of the relationship. The ILFS is highly correlated with physical attractiveness [1]. Physical attractiveness has always proven to be a powerful predictor of mutual attraction and partner choice and is equally applicable to cross-cultural and transgender people [3,4]. The results of Cacioppo et al. [5] indicated that when a person stares at another person’s face for a long time, the first person will subconsciously feel that he/she can develop a long-term relationship with the second person.

Physiological signals are spontaneous responses, i.e., they are not controlled by human consciousness [6]. Therefore, using physiological signals for emotional recognition is a relatively objective method. Previous studies have shown that physiological signals such as those from electrocardiogram, photoplethysmography (PPG), electromyography (EMG), and electroencephalogram contain information related to human emotions [7,8,9,10]. However, it is impractical to measure all physiological signals simultaneously in real life, as this will affect each individual’s personal performance, thereby affecting the quality of experimental data; simultaneously, it will also increase the computational complexity and time. The PPG sensor is one of the most popular sensors in smart watches and wristbands. PPG signal technology is a non-invasive, low-cost technology [11]. PPG signals are widely favored in many applications and measurement methods. Excellent results have been achieved in blood pressure measurement [12], activity recognition [13], heart rate detection [14], sleep quality detection [15], and emotion recognition [16], and great progress has been made in measurement methods. Khan et al. [17] designed an organic multi-channel optoelectronic sensor for wearable health monitoring. Biswas et al. [18] proposed an integrated soft optoelectronics for wearable health monitoring. Hao et al. [19] developed a systematic algorithm for pressure monitoring. Therefore, PPG signals have significant development potential. The acquisition of the PPG signal only requires a sensor to be attached to the index finger of the left hand, and the data can be acquired without affecting the comfort of the person [20]. Therefore, in this study, we acquired and analyzed the PPG signal.

In this study, we conducted experiments and carried out analyses based on the relationship between the ILFS and physical attractiveness, and based on the important roles of physiological signals in emotion recognition. The structure of this paper is as follows. Section 2 introduces the experimental setup. Section 3 introduces the proposed emotion recognition algorithm based on the PPG signal. Section 4 presents the experimental results and discussion. Section 5 presents the conclusions.

## 2. Experimental Setup

### 2.1. Participants

A total of 46 (22 males and 24 females, heterosexual) college students from Southwest University participated in this experiment. Their ages ranged from 17 to 26 years (19.7 ± 1.6). They had no history of medical illness caused by heart disease and/or respiratory or central nervous system disease. After a detailed introduction to the experimental protocol, all participants provided written informed consent. At the end of each experiment, a certain amount of money was paid as a reward to thank them for their participation. The study was conducted with the approval of the Research Ethics Committee of Southwest University.

### 2.2. Emotional Stimulation

In this study, 1000 (500 males and 500 females) background-monotonous, face-toward-camera high-resolution portrait photos were acquired from the Internet. Moreover, Photoshop was used to uniformly crop the pictures into 840 × 1080 pixels size. To select the pictures that could better induce the target emotion, we conducted a preliminary study before the formal experiment. Sixty college students (30 males and 30 females) were shown pictures of the opposite sex, and a nine-level Likert scale was used to evaluate the attractiveness of the pictures, i.e., as high, medium, or low. Ultimately, 480 (240 per male and female) portraits with high, medium, and low attractiveness were selected as emotional stimulation materials.

### 2.3. Experimental Procedure

**Figure 1:**
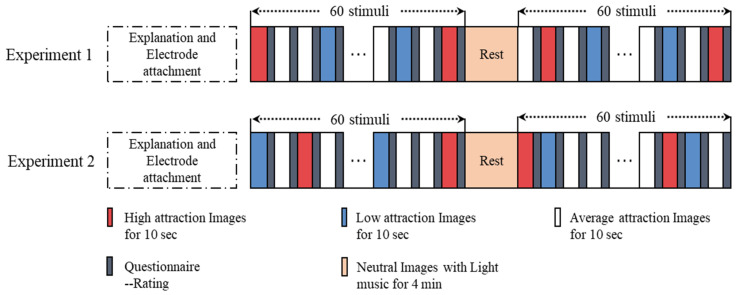
Impulse of love at first sight (ILFS) emotion-induced experimental procedure.

Before the start of the experiment, the participants were introduced to the experimental procedures in detail, and there was an appropriate time to rest and adapt to the experimental environment. To obtain high-quality data, the participants were asked to keep their left hand motionless during the experiment, except for when resting. The experimental procedure is shown in Figure 1. To avoid affecting the quality of the data owing to the long experiment time, each participant was asked to conduct two experiments, each with different emotional stimulation materials. The time interval between the two experiments was 1 day or longer. Each experiment had two sections. Each section contained 60 stimuli, and each stimulus was presented randomly and lasted for 10 s. After the stimulus presentation, the participants were asked to self-report their ILFS based on their feelings. The ILFS ranged from no (0) to very strong (3). The natural pictures were accompanied by light music, and were kept for 4 min between the two sessions. This process allowed the subjects to recover from their respective emotional state to a calm state.

### 2.4. Physiological Signal Recording

A Biopac MP150 (Biopac System Inc., Goleta, California, USA) physiological data acquisition system and AcqKnowledge v4.2 (Biopac, USA) software were used to acquire the PPG signals. The physiological sensor was a module attached to the Biopac physiological data acquisition system. The PPG sensor was attached to the first joint of the index finger of the left hand, and the signal was recorded at a sampling rate of 1000 Hz. Appropriate amplification and band-pass filtering were conducted. The signal collection started 1 min before the emotional stimulus was presented.

## 3. Method

**Figure 2:**
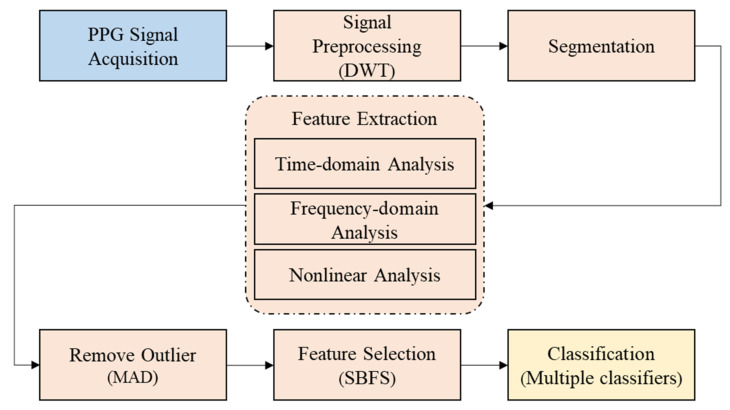
Block diagram of the impulse of love at first sight (ILFS) recognition algorithm.

Figure 2 shows a block diagram of the proposed algorithm for the recognition of the ILFS based on PPG signals. We briefly introduce each process of the block diagram. The steps are as follows:

(1) Signal preprocessing: A discrete wavelet transform (DWT) is used to eliminate noise (such as baseline drift, power frequency interference, and EMG interference) from the original PPG signal.
(2) Segmentation: The preprocessed PPG signal is intercepted according to the 10 s time interval from the appearance to the end of the picture.
(3) Feature extraction: 26 physiological features are extracted from a time domain analysis, frequency domain analysis, and nonlinear analysis of the PPG signal.
(4) Remove outliers: The median absolute deviation (MAD) algorithm is used to remove outliers.
(5) Feature selection: The sequence backward floating selection (SBFS) algorithm is used to select suitable features from the 26 features.
(6) Classification: A variety of classifiers (support vector machine (SVM), random forest (RF), linear discriminant analysis (LDA), and extreme gradient boosting (XGBoost)) are used for emotion recognition.

We introduce the steps of the proposed ILFS recognition algorithm in more detail below.

### 3.1. Preprocessing

A PPG signal measured in a laboratory is easily affected by the monitoring equipment and power supply, as well as by human breathing, limb movement, and temperature changes in the sensors. The original PPG signal is prone to noise and artifacts. This type of noise mainly comprises power frequency interference, EMG interference, and baseline drift.

A wavelet transform is a linear process that can decompose a signal into components of different scales (or resolutions) [21]. It has two types, namely the continuous wavelet transform and discrete wavelet transform (DWT) [22]. The DWT is widely regarded as a key tool for signal analysis [23], signal detection [24], and signal denoising [25]. Denoising technology has been established as a major area of signal analysis for many applications [26]. Therefore, we chose the DWT to denoise the signal. First, the PPG signal was down-sampled to 200 Hz through the AcqKnowledge v 4.2 software. Then, the bior3.5 wavelet was used to decompose the signal with four layers of wavelets, and then a soft threshold was used to threshold the coefficients of each scale to remove high-frequency noise. Then, the signal after the first wavelet denoising was subjected to 8-layer wavelet decomposition. The approximate components on the 8th layer were completely removed, and the other layers were reconstructed to obtain the signal after removing the baseline drift.

### 3.2. Label Processing

In this study, we divided the scores into two categories based on the participants’ self-evaluated ILFS. A score higher than 1 was set to 1 and meant that the ILFS was generated; a score of 0 was set to 0, meaning that the ILFS was not generated. A score of 1 indicated that the participant was in a state of ambiguity in regards to the ILFS; we did not use such data.

### 3.3. Feature Extraction

We extracted four types of features from the preprocessed PPG signal. These were the geometric time-domain features of the PPG signals, time-domain features of the heart rate variability (HRV) from statistical methods, frequency-domain features, and nonlinear features of the HRV from spectrum analysis. All of the features were extracted from the 10 s interval after segmentation. The 26 features extracted from each 10 s of data constituted a feature vector, which was used as the input to the classifier for recognizing the ILFS. The extracted features and their descriptions are shown in Table 1.

**Figure 3:**
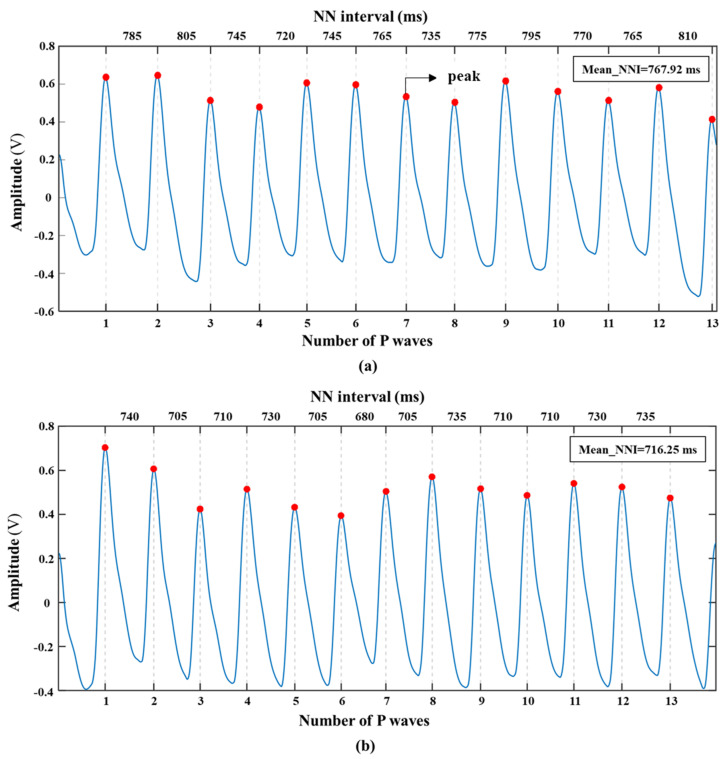
The waveform changes of PPG signal in two emotional states (ILFS was not generated (**a**) and ILFS was generated (**b**)).

Figure 3 shows the waveform changes of PPG signals (peak detection results and NN intervals) of a subject in two emotional states (ILFS was generated and ILFS was not generated).

### 3.4. Remove Outliers

The features extracted from a PPG signal include outliers that affect the performance of the classifier. Therefore, it was particularly important to delete these outliers. The MAD is a simple but effective method for removing outliers [27]. The main idea of this method is to use the median and median deviation, rather than the more commonly used average and standard deviation [28]. First, the distances between all sample values and the median of the sample values are calculated, and the MAD is obtained according to the absolute value of the median of the obtained distances. The MAD and corresponding method of removing outliers are represented in Equations (1) and (2).

(1) $$MAD=1.4826\times M_{i}(abs(x_{i}-M_{j}(x_{j})))$$

(2) $$M(x_{i})-3\times MAD\leq x_{i}\leq M(x_{i})+3\times MAD$$

In the above, *x_j_* represents one of the n sample values, and *M_i_* is the median of the sequence.

### 3.5. Feature Selection

Feature selection plays an important role in the establishment of a classification system [29,30,31,32]. It can not only reduce the dimensionality of the data but can also reduce the amount of calculation and obtain a good classification performance [33]. Feature selection is usually used to select a subset of relevant features from a large number of original features [34]. Irrelevant features not only lead to insufficient classification accuracy, but also increase the difficulty of finding potentially useful information [35,36]. Pudil et al. [37] introduced the concept of a “floating feature search” and two “floating” feature selection methods, i.e., sequence forward floating selection, and SBFS. They can be seen as extensions of the sequence forward selection and sequence backward selection feature selection algorithms. This study used the SBFS feature selection algorithm. The SBFS algorithm is the process of selecting “*k*” optimal feature subsets from “*n*” features. In this study, “*A*” represented the set of all features as follows:

$$A=\{a_{1},a_{2},\ldots ,a_{n}\}$$

The SBFS takes the “*A*” set as input, and the output after feature selection is “*B*” (i.e., a subset of “*A*”). The idea of the algorithm is to train all of the features through the classifier, and then to remove features that reduce the classification accuracy from the set one-by-one. This feature elimination process continues until there are only “*k*” features remaining in the feature vector. Finally, an optimal feature subset “*B*” of length “*k*” is obtained as follows:

$$B=\left\{b_{j}|j=1,2,\ldots ,k;b_{j}\in A\right\}$$

The length “*k*” can be represented as follows:

k=(1,2,3,…,n)

### 3.6. Performance Metrics

In the binary classification problem, the sample value can be divided into four situations, namely true positive (TP), true negative (TN), false positive (FP), and false negative (FN), based on the combination of the true and predicted categories. We used four common indicators, namely F1 score (F1), accuracy (Acc), specificity (Sp), and sensitivity (Se), to evaluate the performance of the classifier. These are statistical measures of the performance of the binary test. They are calculated using Equations (3)–(6), respectively.

(3) $$F1=\frac{2TP}{2TP+FP+FN}\times 100$$

(4) $$Acc=\frac{TP+TN}{TP+FP+FN+TN}\times 100$$

(5) $$Sp=\frac{TN}{TN+FP}\times 100$$

(6) $$Se=\frac{TP}{TP+FN}\times 100$$

## 4. Results and Discussion

In this study, four classifiers (SVM, RF, LDA, and XGBoost) were evaluated. All features were standardized to the range of [−1, 1]. We used four performance indicators, namely F1, Acc, Sp, and Se, along with 10-fold cross-validation to evaluate the classification performance of the four machine learning algorithms with or without feature selection. Table 2 shows the performance comparison obtained without using feature selection.

To obtain the optimal feature subset from the 26 features based on the SBFS feature selection algorithm, we changed the number of features from 1 to 26, randomly selected cross-validated training samples and test samples from the original data set, and performed 10-fold cross-validation. The error rate was selected as the evaluation function, and the calculation method is shown in Equation (7).

(7) $$e=N_{e}/N_{a}$$

**Figure 4:**
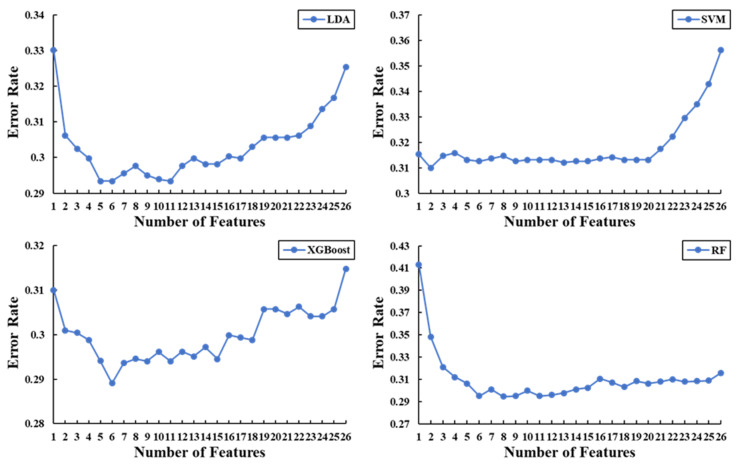
Results of the error rate of the four classifiers with different numbers of features.

Here, *N_e_* and *N_a_* are the number of misclassifications in the test sample and number of all test samples, respectively. Figure 4 shows the variation in the error rate for different numbers of features for different classifiers (with feature selection).

The features corresponding to the minimum evaluation function values of the different classifiers are regarded as the optimal feature subsets of the corresponding classifiers. Table 3 compares the classification performances of the optimal feature subset on the four classifiers for 10-fold cross-validation. These results show that the XGBoost classifier obtained the best classification performance.

**Figure 5:**
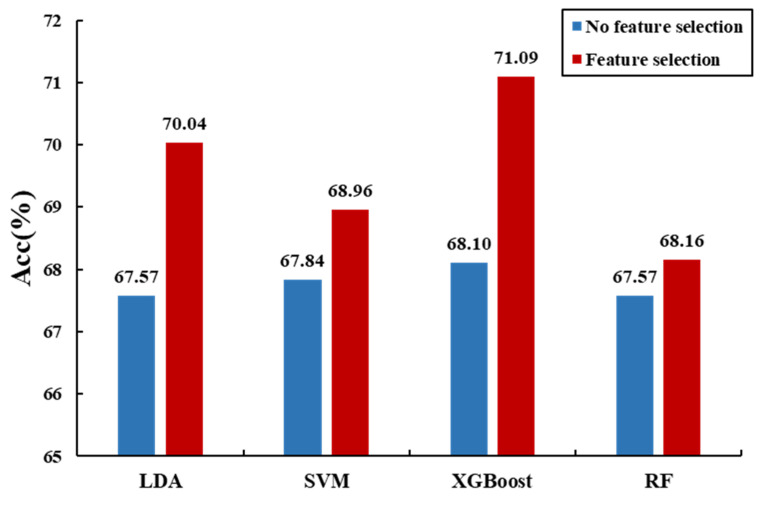
Comparison of classification accuracy of different classifiers without or with feature selection.

In this paper, we proposed a reliable ILFS recognition algorithm, using PPG signals, comprising an SBFS feature selection algorithm and classifier. To study the most effective/optimal classification methods, we used four methods for comparing the machine learning algorithms. Figure 5 shows a comparison of the classification accuracies of the different classifiers with and without feature selection. It can be seen that feature selection uses the least number of features to improve the classification accuracy, thereby reducing the computational cost. Moreover, the method of feature selection (as combined with the XGBoost classifier) is the best approach for recognizing the ILFS emotions relative to the other machine learning algorithms.

## 5. Conclusions

The research in this study shows that features extracted from the time domain, frequency domain, and a nonlinear analysis of PPG signals can provide discriminative information for the ILFS. Simultaneously, it also demonstrates the possibility of emotion recognition based on PPG signals. PPG signals are physiological signals that are widely used in wearable devices and have great commercial value. This research has laid the foundation for the use of wearable devices to recognize a love impulse. In the future, we hope that the integration of algorithms into wearable devices will help increase the success rate of relationships.

## Figures and Tables

**Table 1:** Description of photoplethysmography (PPG) features.

ID	Features	Description
*Time-domain (Geometrical) features*		
1	Mean_HR	Mean of instantaneous heart rate
2	Mean_VP	Mean of the time from Valley to Peak
3	Mean_PV	Mean of the time from Peak to Valley
4	Mean_NNI	Mean of the time from Peak to Peak
5	Mean_VVI	Mean of the time from Valley to Valley
*Time-domain (Statistical) features*		
6	RMSSD	Root mean square of successive differences of NN interval
7	SDNN	Standard deviation of NN interval
8	SDSD	Standard deviation of successive differences of NN interval
9	Range_NN	Difference between the maximum and minimum NN interval
10	NN50	Number of interval differences of successive NN interval greater than 50 ms
11	pNN50	Corresponding percentage of NN50
12	NN20	Number of interval differences of successive NN interval greater than 20 ms
13	pNN20	Corresponding percentage of NN20
14	CVSD	Coefficient of variation of successive differences equal to the RMSSD divided by Mean_NNI
15	CVNNI	Coefficient of variation equal to the ratio of SDNN divided by Mean_NNI
*Frequency-domain features*		
16	LF	Total energy of NN interval in the low frequency band (0.04–0.15 Hz)
17	HF	Total energy of NN interval in the high frequency band (0.15–0.4 Hz)
18	LF/HF ratio	Ratio of LF power to HF power
19	Total_Power	Total energy of NN interval
20	nLFP	Normalized low frequency power
21	nHFP	Normalized high frequency power
*Nonlinear (Geometrical) features*		
22	SD1	Standard deviation for T direction in Poincare plot
23	SD2	Standard deviation for L direction in Poincare plot
24	SD12	Ratio between SD2 and SD1
25	CSI	Cardiac Sympathetic Index
26	CVI	Cardiac Vagal Index.

**Table 2:** Comparison of performance of different classifiers without feature selection.

Classifier	F1 (%)	Acc (%)	Se (%)	Sp (%)
LDA	68.33	67.57	69.97	65.18
SVM	69.59	67.84	73.59	62.09
XGBoost	68.29	68.10	68.69	67.52
RF	68.07	67.57	69.12	66.03

**Table 3:** Comparison of performance of different classifiers with feature selection.

Classifier	F1 (%)	Acc (%)	Se (%)	Sp (%)	Selected Features
LDA	71.98	70.04	75.29	64.76	5, 13, 17, 19, 21
SVM	69.84	68.96	71.88	66.03	16, 17
XGBoost	71.59	71.09	72.84	69.33	4, 9, 13, 14, 15, 17
RF	68.39	68.16	68.90	67.41	3, 4, 9, 10, 17, 22
